# Research Progress on Active Ingredients and Product Development of *Lycium ruthenicum* Murray

**DOI:** 10.3390/molecules29102269

**Published:** 2024-05-11

**Authors:** Ming-Lu Xu, Yun-Feng He, Liang Xie, Ling-Bo Qu, Guang-Ri Xu, Cheng-Xing Cui

**Affiliations:** 1 School of Chemistry and Chemical Engineering, Institute of Computational Chemistry, Henan Institute of Science and Technology, Xinxiang 453003, China; mingluxu0827@outlook.com (M.-L.X.); yunfenghe0620@outlook.com (Y.-F.H.); xieliang1449129616@outlook.com (L.X.); 2 School of Food Science, Henan Institute of Science and Technology, Xinxiang 453003, China; 3 School of Chemistry and Chemical Engineering, Zhengzhou University, Zhengzhou 450001, China

**Keywords:** *Lycium ruthenicum* Murray, extraction and identification methods, bioactivities, applied research

## Abstract

*Lycium ruthenicum* Murray possesses significant applications in both food and medicine, including antioxidative, anti-tumor, anti-fatigue, anti-inflammatory, and various other effects. Consequently, there has been a surge in research endeavors dedicated to exploring its potential benefits, necessitating the organization and synthesis of these findings. This article systematically reviews the extraction and content determination methods of active substances such as polysaccharides, anthocyanins, flavonoids, and polyphenols in LRM in the past five years, as well as some active ingredient composition determination methods, biological activities, and product development. This review is divided into three main parts: extraction and determination methods, their bioactivity, and product development. Building upon prior research, we also delve into the economic and medicinal value of *Lycium ruthenicum* Murray, thereby contributing significantly to its further exploration and development. It is anticipated that this comprehensive review will serve as a valuable resource for advancing research on *Lycium ruthenicum* Murray.

## 1. Introduction

*Lycium ruthenicum* Murray (LRM), a member of the Solanaceae family, is a spiny, branching shrub known for its distinctive white and purplish-black fruit, which results from inhibited anthocyanin synthesis within the plant [1]. LRM exhibits significantly higher phytochemical content compared to *Lycium barbarum* (LB), displaying superior antioxidant activity as well [2]. Its beneficial effects include antioxidative, anti-tumor, anti-fatigue, and anti-inflammatory properties, attributed mainly to its rich array of active ingredients such as polysaccharides, anthocyanins, flavonoids, polyphenols, amino acids, and minerals.

LRM’s therapeutic potential is evidenced by its protective effects, including mitigating alcoholic liver and kidney injury in animal models [3,4]. Li et al. [5] identified anti-inflammatory effects in LRM fruit extracts, potentially mediated by 5-hydroxymethylfurfural. Chemical extraction methods are commonly employed to isolate these active constituents. Polysaccharides, for instance, can be extracted through various techniques including hot water leaching, acid or alkali extraction, and low eutectic solvent extraction. Similarly, polyphenols can be obtained via ultrasonic, microwave, enzyme-assisted, or supercritical liquid extraction methods, among others.

These extracted fractions hold promise for pharmaceutical applications, either in their native form or following modification with functional groups. LRM’s potential as a therapeutic agent extends to conditions like Alzheimer’s disease (AD), with compounds like *N*-acetyl-l-tryptophan possibly targeting calmodulin and exerting anti-AD effects. Moreover, LRM’s multitargeted approach in modulating neurological, immune, and signaling pathways suggests potential efficacy in mitigating AD pathology [6,7].

This review is divided into three main parts: extraction and determination methods, their bioactivity, and product development. The purpose of this study is to provide a comprehensive and systematic summary and classification of the nutritional composition, efficacy, and product development over the past five years, for academic research or practical reference.

## 2. Extraction and Determination Methods

The extraction and determination details of LRM active ingredients are shown in Tables S1 and S2 (Supplementary Information).

### 2.1. Extraction and Identification of LRPs

#### 2.1.1. LRP Extraction

Polysaccharides can be extracted through various methods, encompassing chemical, biological, physical, or hybrid approaches. Among these, the chemical method stands out as the most prevalent, employing techniques such as hot water leaching. Biological extraction relies on enzymatic processes, while physical extraction utilizes instruments like ultrasonic and microwave devices, as well as supercritical CO_2_ extraction.

In traditional methodologies, He et al. [8] utilized hot water leaching to extract *Lycium barbarum* polysaccharides (LRPs), yielding a novel arab galactose LRP1-S2. Li et al. [9] optimized this approach by refining the hot water extraction, ultrasonic-assisted extraction, and enzymatic extraction methods. Their findings indicate that enzymatic extraction yielded the highest LRP content, followed by ultrasonic and hot water methods. Enzymatic extraction favored arabinose, ultrasonic-assisted extraction favored glucose, and hot water extraction yielded trace amounts of sucrose. Notably, hot water extraction exhibited superior extraction of sugar components compared to enzymatic and ultrasonic methods.

Additionally, Bai H. et al. [10] extracted LRPs using the conventional water bath method, ultrasound method, microwave method, and ultrasound-microwave synergistic extraction method. The results showed that the ultrasound–microwave synergistic extraction method > conventional water bath extraction method > microwave extraction method > ultrasound extraction method. In summary, the ultrasound–microwave synergistic extraction method is an effective method for extracting LRPs.

Recent advancements have introduced new extraction techniques. Jin et al. [11] optimized LRP extraction using high-hydrostatic-pressure-assisted extraction (HHPE), investigating its effects on LRP extraction yield. Their findings revealed that the optimal HHPE conditions included a pressure of 380 MPa, an extraction time of 8 min, and a ratio of 25 mL/g of LRM to water. Under these conditions, the polysaccharide yield closely matched predictions, showcasing HHPE as a viable method for LRP extraction. Compared to traditional methods, HHPE proved to be efficient, saving both time and energy, and holds potential for enhancing the extraction efficiency of polysaccharides from various plant sources.

#### 2.1.2. Determination of LRP Content and Composition

In addition to extraction methods, determining content and composition is crucial. Traditional content determination methods include the phenol–sulfuric acid, anthrone–sulfuric acid, and 3,5-dinitrosalicylic acid methods, with the phenol–sulfuric acid method being the most commonly used [12]. Current techniques for analyzing the monosaccharide composition of plant polysaccharides include capillary electrophoresis, gas chromatography, high-performance liquid chromatography, and mass spectrometry [13].

Emerging alongside traditional methods are more convenient approaches. Zhang et al. [14] demonstrated the use of 1H-Nuclear Magnetic Resonance Spectrum (NMR) for rapid and accurate quantitative analysis of LRP. The highest content of LRP in July was 0.22 mg/g. This method offers advantages such as short measurement time, a strong linear relationship, stability, repeatability, and accurate control without sample decomposition.

Zhang [15] used the PMP (1-phenyl-3-methyl-5-pyrazolone) derivation method and the sugar alcohol acetate method combined with HPLC to determine the monosaccharide composition in polysaccharides and found that LRP1-S2 contains mannose, rhamnose, glucuronic acid, galacturonic acid, glucose, galactose, and arabinose. LRP3-S1 contains rhamnose, galacturonic acid, galactose, arabinose, and xylose.

Li et al. [16] established a tandem mass spectrometry method for the simultaneous determination of sucrose, fructose, rhamnose, arabinose, glucose, cottonseed sugar, and mannose in LRM. This method is simple, sensitive, and suitable for LRP determination. Contrasting with traditional methods, which measure content or composition individually, the new method allows simultaneous measurement and offers the advantages of shorter measurement time, precise control, and simplicity. Its application to other plant polysaccharides could significantly save time.

### 2.2. Extraction and Identification of LRM Polyphenols

#### 2.2.1. LRM Polyphenol Extraction

Polyphenols, including tannins and flavonoids such as proanthocyanins and anthocyanins, are prevalent in medicinal plants. Extraction methods for polyphenols include ultrasonic extraction, microwave extraction, enzyme-assisted extraction, supercritical liquid extraction, ion precipitation extraction, and membrane extraction.

Guo et al. [17] optimized the ultrasonic-assisted aqueous two-phase extraction of proanthocyanidins from LRM. Under optimal conditions—ethanol volume fraction of 61%, ammonium sulfate mass concentration of 0.24 g/mL, material-to-liquid ratio of 1:26, ultrasound time of 49 min, and ultrasound power of 220 W—the extraction rate of *Lycium ruthenicum* Murr. proanthocyanidins (LRMPs) reached 6.113%. Wu et al. [18] compared solvent extraction with ultrasonic-assisted extraction of polyphenols from LRM, revealing the latter as a simple, economical, time-saving, low-energy-consumption method with high extraction efficiency. Mi et al. [19] optimized the ultrasonic-assisted pectinase extraction of anthocyanins from LRM using orthogonal experiments, yielding 19.4 ± 0.87 mg/g under the conditions of a 45 °C extraction temperature, 30 min extraction time, 0.5% enzyme content, 360 W ultrasonic power, and pH = 1. Stability experiments demonstrated the preservation of LRM-extracted anthocyanins in low-temperature and high-acidity storage solutions.

#### 2.2.2. LRM Anthocyanins Content Determination

Common methods for determining anthocyanin content include the pH differential method, liquid chromatography, and spectrophotometry, among others.

Liu et al. [20] employed the pH differential method to assess anthocyanin content extracted through various processing methods of LRM. The results indicated the lowest content with spray drying, followed by ultra-fine crushing, notably increasing after purification with macroporous resin and gel chromatography.

Wang et al. [21] utilized high-performance liquid chromatography (HPLC) and UV detection to analyze anthocyanins in LRM extracts. The total anthocyanin content was 2.0 mg Cy/g. Meanwhile, the structure of anthocyanins in LRM was separated and identified by high-performance liquid chromatography–electrospray ionization–mass spectrometry (HPLC-ESI-MS). Two types of anthocyanins have been successfully identified, namely *P*etunidin-3-*O*-rutinoside(glucosyl-p-coumaroyl)-5-*O*-glucoside and *P*etunidin-3-*O*-rutinoside (p-coumaroyl)-5-*O*-glucoside. Gan et al. [22] optimized anthocyanin determination methods by employing ultra-high-performance liquid chromatography–time-of-flight mass spectrometry (UHPLC/FTL) for rapid and precise identification. The average sample recovery rate was 95.45%, with a relative standard deviation of 2.62%. This approach enhances the quality control of LRM due to its superior repeatability and precision compared to conventional methods.

Innovatively, Zhang et al. [23] developed a deep learning approach using near-infrared hyperspectral imaging (NIR-HSI), enabling the simultaneous determination of total phenols, total flavonoids, and total anthocyanins in LRM. Muhammad et al. [24] further refined this technique, optimizing phenolic and flavonoid assays using portable NIR spectroscopy integrated with chemometrics. The resulting models, assessed via calibration and prediction correlation coefficients, root mean square error of prediction, and residual predictive deviation, demonstrated the efficacy of this simple, rapid, and cost-effective approach for quantifying phenols and flavonoids in LRM samples.

### 2.3. Extraction and Composition Determination of LRM Flavonoids

Flavonoids in LRM are predominantly found in leaves and fruit, offering a range of physiological and biochemical benefits such as antioxidant, anti-aging, antibacterial, and lipid-modulating effects. However, research into their specific composition and extraction methods lags significantly behind that of polysaccharides and anthocyanins.

Presently, traditional solvent extraction methods like alcohol and alkali extraction are widely employed, alongside emerging technologies such as supercritical fluid extraction, enzyme engineering, and physical field assistance.

Shu et al. [25] conducted flavonoid extraction from Chaidamu LRM using supercritical CO_2_, followed by analysis using ultra-high-performance liquid chromatography–mass spectrometry and peak view chromatography workstation fitting. Through secondary mass spectrometry analysis, UV spectroscopy, and comparison of mass spectrometry data, they identified 13 flavonoids in the supercritical CO_2_ extraction of LRM, including rutin and *Q*uercetin-3-β-Glucuronide and hyperoside, also known as *Q*uercetin-3-*O*-galactopyranoside, dihydroquercetin, dihydroisorhamnosum, dihydrokaempferol, quercetin, apigenin, hesperidin, alfalfa, kaempferol, isorhamnosum, and kaempferol. Notably, hesperidin and alfalfa were first discovered during the study of LRM components.

### 2.4. LRM Betaine Content Determination

**Figure 1:**
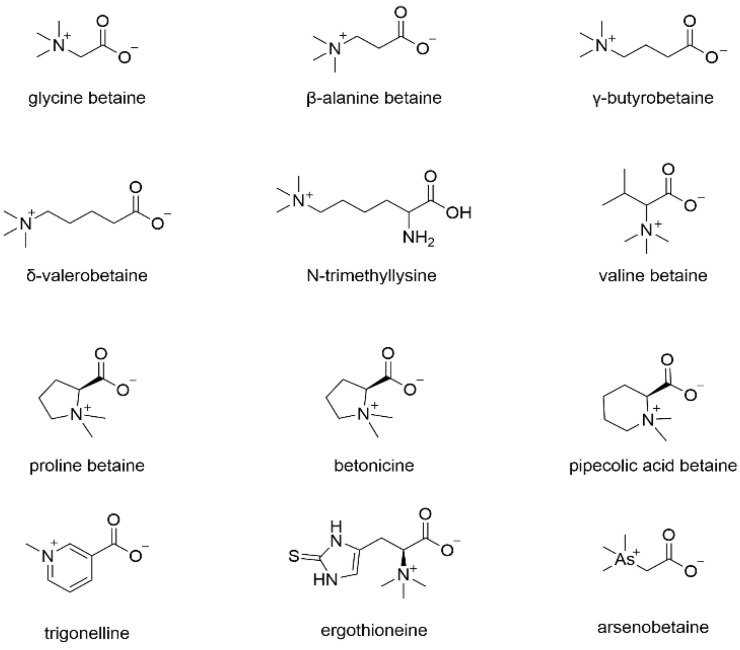
Chemical structure of betaine and its analogs.

Betaine, originally discovered in sugar beets, lends its name to an alkaloid compound. Natural products sharing a chemical connection with betaine are collectively referred to as the “betaine class”. The structural formula of betaine analogs is depicted in Figure 1.

Various methods are employed to determine betaine content, including non-aqueous titration, chemical assays, colorimetry, and gravimetry. Additionally, with technological advancements, HPLC has gained prominence in recent years for betaine quantification.

Currently, research on LRM betaine primarily centers on content detection, with limited exploration into extraction and utilization. Liu et al. [26] utilized reverse-phase (RP)-HPLC to determine the average betaine content in Qaidam LRM, finding it to be 1.34%. Geng et al. [27] employed ion chromatography to assess the betaine content in LRM from different regions, reporting levels as high as 19.58% in Minqin County, Gansu Province, and 9.44% in Golmud City, Qinghai Province.

### 2.5. Extraction and Content Determination of LRM Tannins

Various methods are employed for extracting tannins, including reflux extraction, cellulase hydrolysis, water temperature soaking, ultrasonic extraction, and supercritical CO_2_ extraction, among others.

Commonly utilized techniques for determining tannin content include the leather powder method, potassium permanganate method, casein method, complexometric titration method, colorimetric method, and spectrophotometry.

Research on tannins in LRM remains limited. Meng et al. [28] utilized the homogenization method to extract tannins from Xinjiang LRM, determining the average tannin content in LRM through UV–visible spectrophotometry to be 2.47%.

### 2.6. Extraction, Composition, and Content Determination of LRM Fatty Acids

Various techniques are commonly employed for fatty acid extraction, including ultrasound-assisted extraction, Soxhlet extraction, supercritical fluid extraction, accelerated solvent extraction, microwave-assisted extraction, and solid-phase extraction.

Traditional methods for determining fatty acids primarily involve chromatography and chromatography–mass spectrometry. Rapid detection technologies such as NIR, NMR, and Raman Spectroscopy (RS), combined with chemometrics and hyperspectral imaging, are also utilized. Additionally, direct ionization techniques like Desorption Electrospray Ionization–Mass Spectrometry (DESI-MS), Extractive Electrospray Ionization Mass Spectrometry (EESI), and Internal Extractive Electrospray Ionization (iEESI)–MS, coupled with multivariate statistical methods, are gaining prominence.

In a study by Yossa Nzeuwa I.B. et al. [29], LRM was extracted with petroleum ether using a Soxhlet apparatus for 2 h. Analysis of the extracted oils via gas chromatography (GC)–MS revealed major fatty acid components including linoleic acid (59.38%), oleic acid (20.85%), palmitic acid (7.07%), linolenic acid (2.98%), and stearic acid (5.31%). Using inductively coupled plasma–atomic absorption spectrometry (ICP-AAS), the main mineral nutrients in LRM were identified as potassium, calcium, and magnesium, with traces of copper, iron, manganese, and zinc. Research on LRM fatty acids remains limited, and the mentioned measurement methods offer valuable insights for future investigations into LRM fatty acids.

### 2.7. Extraction and Determination of Phenylpropanoid Derivatives

Various extraction methods for phenylpropanoid include water extraction, organic solvent extraction, microwave-assisted extraction, and ultrasonic-assisted extraction, among others.

Methods for determining phenylpropanoid include ultraviolet spectrophotometry, second derivative spectroscopy, visible spectrophotometry, HPLC, RP-HPLC, solid-phase extraction microcolumn high-performance liquid chromatography (SPE-MHPLC), and others.

**Figure 2:**
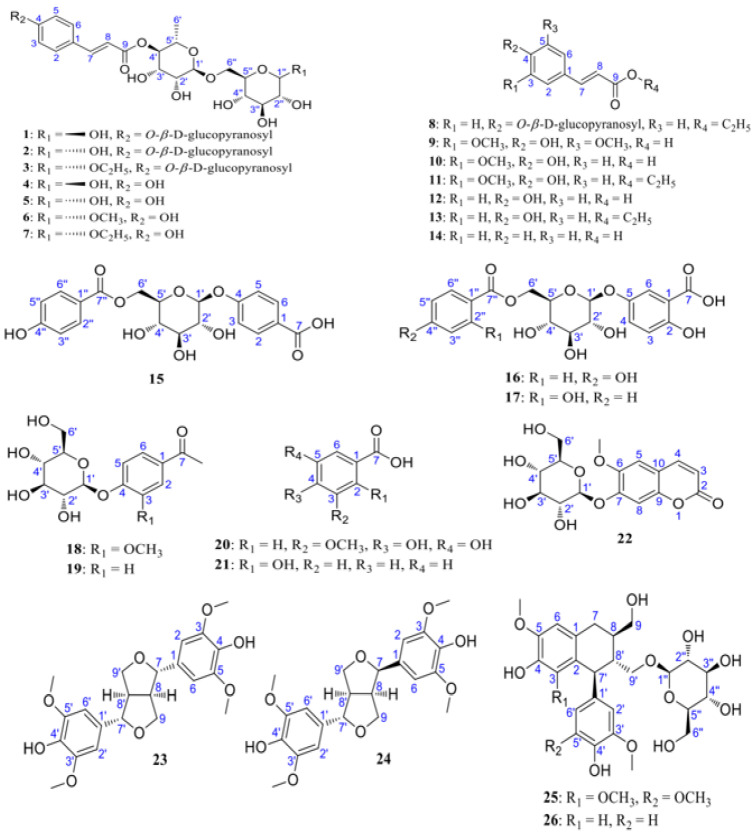
Chemical structures of compounds **1**–**26**. Reprinted with permission from reference [28], copyright 2022 Elsevier.

In a study by Zhao et al. [30], LRM was extracted using ethanol and condensed. Grades A–D were obtained through continuous elution of the concentrated solution via an HP-20 macroporous resin column. HPLC was employed to analyze the components, and similar compounds were further isolated using open silica CC, resulting in the identification of 26 compounds. Among these, 8 were identified as new phenylpropanoid derivatives (**1**–**7** and **15**), while the remaining 18 were known derivatives (**8**–**14**, **16**–**26**). Notably, compounds **16**, **17**, **24**, and **26** were isolated from *Lycium* for the first time (detailed information is shown in Figure 2).

### 2.8. Extraction and Determination of Polyphenol Glycosides

Traditional extraction methods for polyphenolic glycosides typically involve impregnation and solvent extraction. However, with advancements in modern technology, researchers have enhanced and optimized these traditional methods, introducing newer techniques such as ultrasonic extraction and microwave-assisted extraction.

Determining polyphenolic glycosides necessitates a combination of various analytical methods, including chromatography, mass spectrometry, and Nuclear Magnetic Resonance.

**Figure 3:**
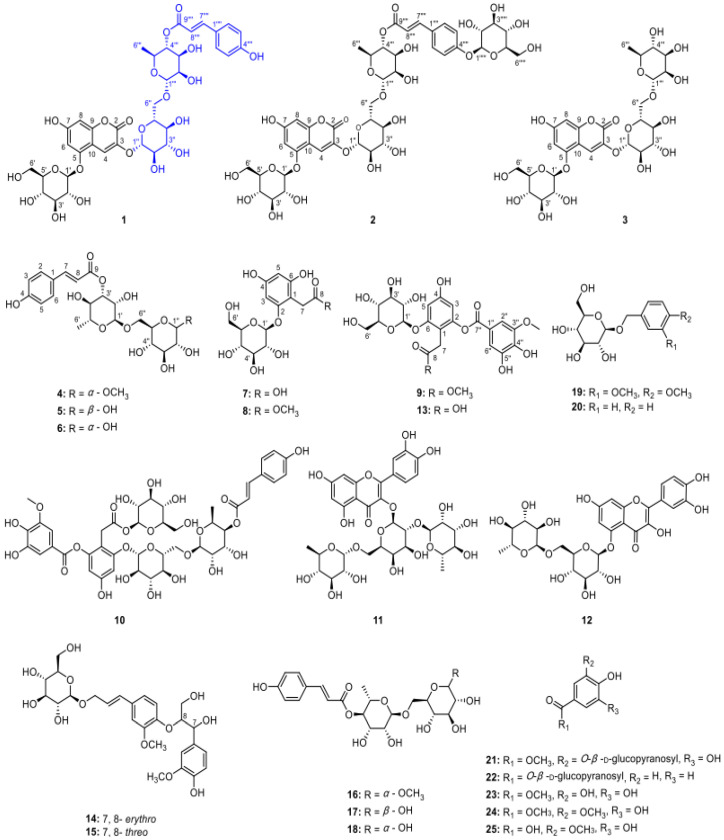
Structures of compounds **1**–**25** obtained from LRM. Reprinted with permission from reference [29], copyright 2022 American Chemical Society.

In a study conducted by Hu et al. [31], polyphenolic glycosides were extracted using a solvent method and analyzed using high-resolution electrospray ionization–mass spectrometry (HRESIMS) and comprehensive NMR analysis. Through chemical hydrolysis, nine new polyphenol glycosides were isolated and identified, named lyciumserin A–I (**1**–**9**), along with 16 known compounds (**10**–**25**). Remarkably, compounds **1** and **2** exhibited significant neuroprotective effects in a PC12 cell model of injury induced by 6-hydroxydopamine. The identified compounds (25) include a variety of glycosides such as 7-hydroxycoumarin-3-*O*-[6-*O*-(4-*O*-p-trans-coumaroyl)-α-l-rhamnopyranosyl]-β-d-glucopyranosyl-5-*O*-β-d-glucopyranoside (**1**), 7-hydroxycoumarin-3-*O*-[6-*O*-(4-*O*-(4-*O*-β-d-glucopyranosyl)-trans-p-coumaroyl)-α-l-rhamnopyranosyl]-β-d-glucopyranosyl-5-*O*-β-d-glucopyranoside (**2**), and others as listed in Figure 3.

## 3. Bioactivities

**Figure 4:**
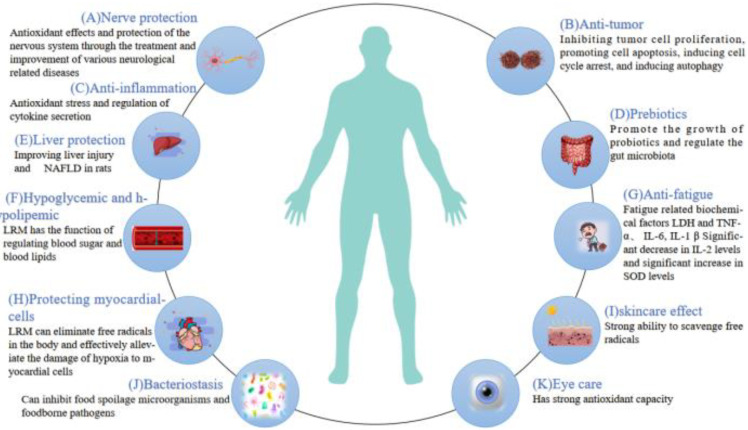
Biological activity of LRM extract. (**A**) Nerve protection; (**B**) anti-tumor; (**C**) anti-inflammation; (**D**) prebiotics; (**E**) liver protection; (**F**) hypoglycemic and hypolipemic; (**G**) anti-fatigue; (**H**) protecting myocardial cells; (**I**) skin care effect; (**J**) bacteriostasis; (**K**) eye care.

LRM is rich in bioactive compounds, making it a promising candidate for developing various products (as summarized in Table S3 (Supplementary Information) and illustrated in Figure 4).

### 3.1. Anti-Tumor Activity

Polysaccharides and anthocyanins found in LRM exhibit potent anti-tumor properties. LRP1-S2, a novel polysaccharide extracted from LRM, can inhibit the proliferation of pancreatic cancer cells in vitro and in vivo, and it has no obvious toxicity to normal pancreatic HPDE 6-C7 cells and LO2 hepatocytes. It may be achieved by blocking P38 MAPK/NF-κB, and the GSK-3 β/β-Catenin signaling pathway induces the apoptosis of human pancreatic cancer BxPC-3 cells [8]. LRM anthocyanins effectively inhibit the proliferation and migration of human liver cancer HepG2 cells by upregulating proliferation factors (LATS1, LATS2, and MOB1) and autophagy factors (Beclin-1, LC3-II, AMPK, p-AMPK, and LC3-II), and downregulating the mRNA levels of the proliferation factor YAP, the protein expression of the autophagy factor p-mTOR, and the cell cycle factor CDK4 [32,33].

Qin X. et al. [34] found that LRP and LRM anthocyanidins alone (incubated for 48 h) failed to prevent tumor cell proliferation, but once applied together, significant anti-tumor activity was observed, resulting from a combination of LRP and LRM anthocyanidins, this inhibition of cancer cell proliferation occurs by blocking the G0-G1 cell cycle and inducing apoptosis of human colorectal cancer LoVo cells via a reactive oxygen species (ROS)-dependent pathway.

Wang et al. [35,36] found through their research that LRM anthocyanin Pt3G can inhibit the proliferation of prostate cancer DU-145, LNCaP, and PC-3 cells by inducing cell apoptosis. The mechanism of action may be related to the activation of the ROS/PTEN/PI3K/Akt/caspase pathway.

Additionally, LRM’s total flavonoids induce HepG-2 liver cancer cell cycle arrest in the S phase, causing cell division and proliferation to stop. At the same time, apoptosis of HepG-2 liver cancer cells can be induced by upregulating Bax and Casp-3 and downregulating the expression level of Bcl-2 [37].

### 3.2. Prebiotic Effects

LRM polysaccharides serve as prebiotics, promoting the growth of *Lactobacillus*, *Bifidobacterium*, *Streptococcus thermophilus* G2, and *Lactobacillus paracasei* L9, and inhibiting the growth of *Enterobacterium* and *Enterococcus* [38,39,40]. They are fermented in the colon to produce short-chain fatty acids, which contribute to gut health and overall well-being. LRM anthocyanins have also been shown to modulate gut microbiota diversity and metabolic pathways, offering potential benefits for obesity and metabolic disorders [41]. Their mechanism of action may be related to increasing the diversity of the mouse gut microbiota, especially reducing the proportion of Firmicutes/Bacteroidota. On the other hand, it may be related to differences in gene expression in metabolic pathways or PPAR signaling pathways.

### 3.3. Anti-Fatigue Properties

LRM polysaccharides and tea polyphenols (TPs) exhibit anti-fatigue effects by reducing oxidative stress and inflammation [42,43]. Changes in fatigue-related biochemical factors lactic acid dehydrogenase (LDH), superoxide dismutase (SOD), tumor necrosis factor-α (TNF-α), interleukin-1β (IL-1β), interleukin-2 (IL-2), and interleukin-6 (IL-6) were measured in motor fatigue (EIF) mouse models treated with TPs and LRPs. The results showed that TP and LRP both showed significant anti-inflammatory effects and decreased oxidative stress. Compared to the control group, LDH, TNF-α, IL-6, IL-1β, and IL-2 levels were significantly reduced and SOD levels were significantly increased in the TP- or LRP-treated group. The results showed that TPs and LRPs were effective in the treatment of exercise fatigue. These compounds improve energy metabolism and mitigate fatigue-related biochemical changes, offering potential as natural remedies for combating fatigue.

### 3.4. Eye and Skin Care Benefits

LRM components, particularly polysaccharides and anthocyanins, show promise in eye and skin care. LRPs can enhance the antioxidant capacity of ARPE-19 cells, inhibit the apoptosis of ARPE-19 cells, and protect retinal pigment epithelial cells from oxidative stress. In addition, purified LRPs can enhance the antioxidant capacity of ARPE-19 cells without dose dependence. They can increase the activities of superoxide dismutase (SOD) and glutathione peroxidase (GSH Px) in ARPE-19 cells damaged by H_2_O_2_, reduce the content of malondialdehyde (MDA), and downregulate NLRP3, caspase-1, and IL-1β. The expression of proteins inhibits ARPE-19 cell apoptosis, thereby preventing age-related macular degeneration [44,45,46,47].

Additionally, LRM anthocyanins reduce the cell apoptosis rate through the death receptor pathway, decrease the expression of TNF-α and caspase-7, and increase the expression of survival proteins, thereby exerting a protective effect on human skin fibroblasts (HSFs) exposed to UVB [48,49]. These properties highlight the potential of LRM-derived compounds in promoting eye and skin health.

### 3.5. Neuroprotection

Neurological diseases often involve cellular damage caused by oxygen free radicals. Polysaccharides found in LRM exhibit antioxidant properties, protecting the nervous system from oxidative stress and apoptosis. LRP3, a component of LRM, has shown promising neuroprotective effects against oxidative stress and apoptosis in rat cortical neurons, suggesting its potential therapeutic role in neonatal hypoxic–ischemic encephalopathy (HIE) [50]. LRM anthocyanins significantly increased the autophagic flow of SH-SY 5Y cells exposed to oxygen–glucose deprivation (OGD), inhibited oxidative stress, reduced the inflammatory response, and decreased neuronal apoptosis. These effects enhance autophagy agonists and weaken autophagy inhibitors. Reducing OGD-induced damage by increasing autophagic flow may be a potential mechanism for anthocyanin protection or treatment of hypoxia and ischemia. Meanwhile, LRM anthocyanins (Pn3G5G) can alleviate cognitive impairment, reduce cortical and hippocampal neuronal loss, lower levels of age-related markers (advanced glycation end products (AGEs) and MDA), increase the ratio of oxidized/reduced glutathione, and inhibit hippocampal neuroinflammation. Pn3G5G can also regulate the biosynthesis of ABC transporters, protein digestion, and cofactors to prevent abnormal neuronal energy metabolism [51,52]. Anthocyanins present in LRM can penetrate the blood–brain barrier and protect nerve cells from damage. Studies indicate that LRM anthocyanins enhance autophagy and reduce oxidative stress, inflammation, and neuronal apoptosis, thereby protecting against hypoxia, ischemia, and age-related neuronal degeneration.

### 3.6. Anti-Inflammatory Effects

Inflammation is implicated in various diseases, including diabetes, Alzheimer’s disease, and arthritis. LRM anthocyanidin can reduce MDA and increase antioxidant levels in H9c2 rat cardiomyocytes by increasing the activity of catalase (CAT), GSH-Px, SOD, and heme oxygenase-1 (HO-1). The energy metabolism of cardiomyocytes under hypoxia was enhanced by increasing Na^+^-K^+^-ATP and Ca^2+^-ATP enzyme activity. By reducing the levels of the pro-inflammatory factors TNF-α and IL-6, increasing the levels of the anti-inflammatory factor IL-10, and inhibiting hypoxia-induced inflammation, H9c2 rat cardiomyocytes were protected from hypoxic damage [53]. Additionally, LRM anthocyanins have been found to alleviate acute gouty arthritis. LRM anthocyanidins inhibit abnormal proliferation and invasive synovial fibroblasts (SFs) in rheumatoid arthritis (RA) patients. In vitro assays confirmed that LRM anthocyanidins inhibit RASF growth through cell cycle arrest and cell invasion paralysis (c-Myc/p21/CDK2) and initiation of apoptosis (HIF-1α/CXCR4/ Bax/Bcl-2), and that LRM anthocyanidin selectively inhibits RASF proliferation without the immune-suppressive side effects normally associated with MTX (methotrexate) treatment of RA, suggesting their potential as natural remedies for autoimmune diseases [54,55].

### 3.7. Hepatoprotection

The liver plays a crucial role in metabolism, and its health is vital for overall well-being. LRPs, a component of LRM, have been shown to protect against liver injury induced by acrylamide poisoning in rats [56]. Moreover, proanthocyanidins from LRM have demonstrated hepatoprotective effects against non-alcoholic fatty liver disease (NAFLD) induced by a cholesterol-rich diet. LRM anthocyanins significantly reduced the accumulation of triglycerides in the liver, reduced inflammation, increased GSH Px activity, and decreased MDA levels. At the same time, it was found that LRM anthocyanins reduced ROS production and inflammation, increased fatty acid oxidation, and reduced fatty acid synthesis in the liver, thereby improving the development of NAFLD induced by a high Western diet. Therefore, oxidative stress and inflammation are also involved in the pathogenesis of NAFLD induced by a Western diet, and they are not solely associated with obesity. There is currently no effective drug or method to treat NAFLD. LRM contains water-soluble antioxidant anthocyanins, which can effectively improve NAFLD, suggesting its potential in preventing and treating NAFLD [57].

### 3.8. Bacteriostasis

Plant extracts rich in polyphenols exhibit natural antimicrobial properties, inhibiting various food spoilage microorganisms and foodborne pathogens. Anthocyanins derived from LRM have been shown to inhibit the growth of foodborne pathogens such as *Staphylococcus aureus* (*S. aureus*), *Escherichia coli* (*E. coli*), *Aspergillus*, and *Penicillium*. The antimicrobial effect varies among these pathogens, with *S. aureus* being the most susceptible, exhibiting a minimum antimicrobial concentration (MIC) of 3.125 mg/mL. Anthocyanins likely exert their antibacterial effects through mechanisms related to membrane permeability and integrity [58,59].

### 3.9. Cardioprotective Effects

Anthocyanins, water-soluble flavonoid pigments with potent antioxidant properties, have been found to mitigate myocardial cell damage induced by hypoxia. In vitro studies using H9c2 rat cardiomyocytes demonstrate that LRM anthocyanins promote cardiomyocyte proliferation and protect against hypoxic-induced cellular damage [60,61,62]. Investigation into the protective mechanisms revealed the involvement of circRNA-related regulatory networks, implicating novel circRNA-miRNA-mRNA interactions in cardiomyocyte protection. Novel_circ_0001555 in LRM anthocyanins may indirectly upregulate the expression of G protein signaling regulator 8 and dystrophin by targeting the sponge adsorption of rno-miR-30b-5p, thereby regulating the calcium signaling pathway, maintaining Ca^2+^ homeostasis, and ultimately protecting hypoxia-induced H9c2 rat cardiomyocytes [63].

### 3.10. Hypoglycemic and Hypolipemic Effects

LRM anthocyanins can promote the phosphorylation of IRS2 and Akt proteins, activate the P13K signaling pathway, promote downstream GSK3 β and FoxO1 phosphorylation, and play a role in inhibiting their activities, thus promoting glycogen synthesis, accelerating sugar consumption, and reducing gluconeogenesis to alleviate the insulin resistance of Hep-G2 cells, showing hypoglycemic and lipid-lowering effects [64]. Meanwhile, LRM anthocyanins bind to pancreatic lipase and inhibit its activity, thereby reducing the hydrolysis of dietary triglycerides and lipid uptake, which in turn plays a role in weight loss [65]. Additionally, the total flavonoid extract of LRM regulates lipid metabolism, improves antioxidant capacity, and significantly reduces the serum total cholesterol (TC), triglyceride (TG), and low-density lipoprotein (LDL-C) concentrations in the serum and liver tissue of hyperlipidemic Sprague Dawley (SD) rats, The concentration of high-density lipoprotein (HDL-C) in serum and liver tissue increased, indicating that the total flavonoids of LRM have the effect of regulating blood lipids [66].

### 3.11. Other Bioactivities

LRM exhibits various biological activities beyond those previously mentioned, including anti-influenza, anti-anxiety, and anti-aging properties, osteoporosis prevention, and gout relief. Kurskaya O. et al. [67] discovered that LRPs possess antiviral properties against influenza A/H3N2 virus, suggesting their potential as novel antiviral agents. Research by Luo J. [68] indicates that LRM anthocyanins alleviate withdrawal symptoms in nicotine-addicted mice, exhibiting anti-anxiety effects and reducing cravings. Xiong L. et al. [69] found that LRM and *Lycium barbarum* (LB) extracts contain phenols and flavonoids that promote longevity by enhancing physiological functions and stress tolerance in nematodes. Wang S.Q. et al. [70] identified a polysaccharide (LRP-S2A) from LRM that promotes osteoblast differentiation, potentially preventing osteoporosis. Li L. et al. [71] investigated the mechanism of LRM anthocyanins in treating gouty arthritis, identifying potential targets and pathways through tissue lipidomics, cyberpharmacology, and molecular docking. Guo S. et al. [72] demonstrated that LRM anthocyanins mitigate mitochondrial damage and autophagy induced by chromium (VI) in cells, suggesting protective effects against heavy metal toxicity.

## 4. LRM Product Development

The applications of LRM extract are shown in Table S4 (Supplementary Information).

### 4.1. LRM’s Product Development in the Field of Packaging Technology

Smart tags and new packaging films are important components of modern packaging technology, providing additional value and functionality to products by integrating advanced materials and sensor technology. These innovative films, characterized by their biodegradability and renewability, provide potential solutions for environmental and health issues related to traditional plastic packaging. Smart tags can serve as indicators of the presence of specific substances in a product or its packaging, reaction signals between different substances, or concentrations of certain components to reflect the quality status of the product. Usually, these indicators are expressed through visual changes. An intelligently active packaging film incorporating LRM anthocyanins was developed. The findings suggest that anthocyanin-rich membranes should be refrigerated to delay degradation and maintain functionality [73].

To enhance the stability of LRM anthocyanins and mitigate oxidative degradation, a gelatin/polyvinyl oxide/LRM proanthocyanidin (GEL/PEO/LPC) fiber membrane was fabricated via electrospinning technology. Fiber membrane encapsulation effectively bolstered the photothermal stability of LRM proanthocyanidin, with higher encapsulation efficiency correlating with stronger antioxidant activity [74].

Xu F. et al. [75] engineered food packaging films by incorporating LRM polyphenols into a starch/polyvinyl alcohol mixture. The resulting film exhibited enhanced compactness and reduced light transmittance, water vapor transmittance, and oxygen transmittance. Moreover, the water contact angle, tensile strength, antioxidant, and antibacterial activities of the membrane were elevated. Notably, LRM extract induced significant color changes in the film across different buffer solutions and ammonia atmospheres, signifying promising prospects for food packaging applications.

LRM anthocyanins exhibit sensitivity to changes in meat food freshness, particularly reacting to ammonia gas, with reaction rates escalating alongside concentration increases. Notably, the color of the anthocyanin solution transitions from fuchsia to cyan to yellow. Additionally, anthocyanins undergo reactive discoloration in response to pH variations. Utilizing a straightforward approach, intelligent labels can be affixed to filter substrates to enable the swift, non-destructive testing of meat product freshness within packaging, thereby safeguarding consumer health [76].

In light of this, LRM anthocyanins were immobilized onto porous filter paper to fabricate LRM anthocyanin paper smart labels. These labels provided real-time and precise feedback on freshness, as demonstrated in subsequent fish preservation experiments. Furthermore, a novel pH-sensitive membrane gel incorporating Artemisia Sphaerocephala Krasch (ASKG), Soybean Protein Isolate (SPI), and LRM anthocyanins was developed. This membrane, prepared by impregnating solid ASKG and SPI substrates with anthocyanidin from LRM dissolved in an acidified alcohol solution, serves as an indicator for monitoring meat freshness during storage [77,78].

Subsequent research unveiled that derivatives from morning glory can chelate with metal ions such as Al^3+^ and Fe^3+^, enhancing color stability across neutral and alkaline pH values. Purple potatoes and LRM, abundant in morning glory derivatives, emerge as promising natural colorant sources applicable over a wide pH range. Through metal chelation, these pigments offer vibrant purples, blues, and greens with heightened stability. They find utility in neutral to slightly alkaline pH food products like milk, milkshakes, ice cream, and creams. This innovation holds significance for the food industry, facilitating the adoption of natural colorants in line with clean labeling trends [79].

### 4.2. Product Development of Plant Growth Regulators

LRM significantly boosts antioxidant levels in the mycelial cells of *Agaricus bitorquis* (*Quél.*) *Sacc* (ABSC), amplifying biomass and polysaccharide production [80]. Wu S. et al. [81] found that using LRM anthocyanins as an inducer notably influenced the liquid fermentation of ABSC in large mushrooms, positively impacting ABSC extracellular polysaccharide (EPS) production and mycelial biomass during fermentation.

### 4.3. LRM in Product Development of Food and Health Drugs

In recent years, LRM has emerged as a notable agricultural product, capturing consumers’ attention and gaining traction in the market due to its status as a nutritious small berry. Consequently, research on LRM processing technology is steadily advancing. However, the majority of LRM and its derivative products’ development has yet to reach its full potential. Key LRM products include chewable tablets [82,83], composite beverages [84,85,86,87], yogurt [88], fruit vinegar [89], and more.

### 4.4. LRM Product Development in the Field of Materials Science

Microcapsule technology employs natural or synthetic polymer materials to encapsulate solids, gases, and other substances into small particles. This technique effectively shields the encapsulated substances from external factors, thereby preserving their activity and extending storage time. Shen M. [90] conducted experiments on LRM anthocyanins microcapsules and identified optimal preparation conditions: embedding time of 66 min, addition of chitosan (CS) at 0.2 mg/mL, and addition of hydroxypropyl-β-cyclodextrin (HP-β-CD) at 1.93 mg/mL. These conditions resulted in an encapsulation rate of 84.28 ± 0.34% and a tyrosinase inhibition rate of 50.75 ± 0.78%. Cell experiments demonstrated that microcapsules significantly enhanced anthocyanins’ inhibition of tyrosinase activity and melanin content in B16 melanoma cells in mice.

### 4.5. LRM’s Product Development in the Textile Industry

Wool, a vital raw material in the textile industry, boasts numerous advantages such as softness, smoothness, elasticity, moisture absorption, and warmth retention. Exploring the use of natural dyes in wool textiles can pave the way for new environmentally friendly products with health functions. Gu J. et al. [91] extracted LRM using an ethanol/water solution and deionized water. Subsequently, the wool fabric was dyed through immersion to produce multifunctional LRM-dyed wool fabric with vibrant colors. The influence of dyeing time on wool fabric dyeing properties was examined, and the fabric’s antioxidant and antibacterial properties were assessed and compared. The results indicated that using an ethanol/water solvent yielded LRM extract with higher anthocyanin content, resulting in deeper surface penetration and stronger antioxidant and antibacterial properties in the dyed wool fabric compared to water alone.

## 5. Conclusions

This article systematically reviews the extraction and content determination methods of active substances such as polysaccharides, anthocyanins, flavonoids, and polyphenols in LRM over the past five years, as well as the determination methods of some active ingredient compositions, biological activities, and product development. These active ingredients have significant benefits for human health, such as anticancer, anti-fatigue, and neuroprotective effects. Through scientific research and product development, these nutrients can be transformed into various healthy foods, health products, drugs, or materials to meet market demand, providing reference values for the application of LRM. But in the past five years, research on LRM has mainly focused on polysaccharides and anthocyanins, while research on flavonoids, amino acids, minerals, and tannins is relatively scarce. Most of the research focuses on the extraction and determination of components, without further investigation into their activity and exploration of their mechanisms. In addition, LRM product development is still relatively shallow, only at the laboratory stage, which limits the exploration of its efficacy and results in the scarcity of commercial products.

However, there are several research issues that urgently need to be addressed, which are also key to LRM product development. Although LRM is considered to have multiple health benefits, current research on its pharmacological effects is relatively limited and lacks in-depth scientific research support. At the same time, research on the pharmacological effects of LRM has been limited to animal experiments, with limited progress in clinical applications. The variety of processed products of LRM is limited, and their market awareness is not high, especially the lack of comprehensive exploration of their auxiliary functions. Therefore, it is necessary to strengthen research on the pharmacological activity of LRM and provide a scientific basis for their application in the fields of medicine and health food. Simultaneously developing diversified processed products, as well as strengthening market promotion and brand building, will help to increase the added value and market share of products.

## Data Availability

No new data were created.

## Supplementary Materials

The following supporting information can be downloaded at: https://www.mdpi.com/article/10.3390/molecules29102269/s1, Table S1: LRM active ingredient extraction; Table S2: LRM active ingredient determination; Table S3: Biological activity of LRM extract; Table S4: Product development of LRM extract.
