# Supplementary material for: Research Progress on Active Ingredients and Product Development of Lycium ruthenicum Murray
Source: Molecules. 2024 May 11;29(10):2269. doi: 10.3390/molecules29102269 (PMC11123928; doi:10.3390/molecules29102269)
Supplement: Supplementary file 1 [file molecules-29-02269-s001.zip › molecules-2950965-supplementary.pdf]

Table S1. LRM active ingredient extraction

Extract	Extraction method	Extraction parameters	Extraction rate	References
Polysaccharide	Hot water extraction	Solid-liquid ratio 1:6.7, Temperature 100℃, Time 4h, Frequency 5	3.11%	[8]
		Solid-liquid ratio 1:27, temperature 90℃, time 60min,	12.3%	
	Ultrasonic-assisted method	solid-liquid ratio 1:21, time 30min, power 200 W	16.1%	[9]
	Enzyme extraction method	Solid-liquid ratio 1:20, PH3.5, temperature 50℃, time 60 min, enzyme amount 0.48%,	20.3%	
	High hydrostatic pressure-assisted extraction	Solid-liquid ratio 1:25,Pressure 380MPa , time 8 min	(8.35±0.12)%	[11]
Proanthocyanidins	Ultrasonic-assisted dual-liquid phase extraction	Solid-liquid ratio 1:26, ammonium sulphate mass concentration of 0.24 g/mL, time 49 min, power 220 W.	6.113%.	[17]
Polyphenols	Solvent method	Solid-liquid ratio 1:50, 1:26Ethanol content was 70%, temperature 58°C, time 37 min.	3.59%	[18]
	Ultrasonic-assisted method	Solid-liquid ratio 1:50, ethanol volume fraction 60%, temperature 36°C, time 31 min, power 240 w.	3.96%	
Anthocyanins	Ultrasonic assisted pectinase extraction	Solid-liquid ratio 1:30, mperature 45 ℃, time 30 min, the enzyme content was 0.5%, power 360 W, pH 1.	19.4%	[19]
Flavonoids	Supercritical CO2 extraction	extraction pressure is 35 MPa, temperature 45°C, time 1h, outlet valve temperature is 110℃		[25]
Tannin	Homogenate method	Solid-liquid ratio 1:30, 70% acetone aqueous solution		[28]
Fatty acids	Soxhlet extraction	Extracted for 2 hours using n-hexane as a solvent		[29]
Phenylpropanoid Deriva-tives	Organic solvent extraction	Solid-liquid ratio 1:5，60% EtOH－H2O，time 2h, Frequency 3		[30]
Polyphenol glycosides	Organic solvent extraction	Solid-liquid ratio 1:8，80% EtOH－H2O，time 3Day.	20%	[31]

Table S2. LRM active ingredient determination

Determination of samples	Measurement meth-od	Conditions	Analysis conditions	Measure advantages	References
Polysaccharide	1H-NMR	Solvent: heavy water	Relaxation time (D1): 1 s, pulse width (P1): 14.90 s, num-	Analysis is simple, fast, and accurate, with short meas-	[14]

			ber of sample scans (NS): 32	urement time	
	UPLC-MS/MS	Chromatographic column: Acquity BEHC18<br>Mobile phase: 0.1% ammonia water, 0.1% ammonia water acetonitrile<br>Electric spray ion source, negative ion mode scanning	Column temperature: 35 ℃, injection volume: 3.0 μ L. Flow rate: 0.3 m L/min<br>Ion source temperature: 150 ℃, desolvent gas: 800 L/h, dry gas temperature: 400 ℃, cone hole gas: 50 L/h, capillary voltage: 2.5 kV	Simple and sensitive	[16]
	pH differential method	PH=3 phosphate buffer solution	2 mL sample solution+18 mL buffer solution	Fast, accurate, simple and easy to implement, without the need for standard samples	[20]
	HPLC-UV	Chromatographic column: Pntulips SBP-C18<br>Mobile phase: methanol, 0.5% formic acid aqueous solution<br>Electrospray ionization, positive ion scanning	Column temperature: 30℃, injection volume: 10 μL, flow rate: 0.8 mL/min,, detection wavelength: 525 nm .	Simple, sensitive, and fast	[21]
Anthocyanins	UHPLC/FTL	Chromatographic column: Acquity UPLC BEHC18<br>Mobile phase: 0.1% trifluoroacetic acid -10% formic acid solution, formic acid water methanol acetonitrile (1:5:2:2, V/V)<br>Electric spray ion source	Column temperature: 25 ℃; Injection volume: 2 μ L. Flow rate: 0.3 mL/min<br>The primary mass spectrometry parameters are within the mass scanning range of m/z: 50 to 1700; Atomization gas pressure: 50 psi; Air curtain pressure: 35 psi; Ion source voltage: 5000 V; Ion source temperature: 500 ℃; Cluster removal voltage: 100 V; Collision energy: 10 eV. The secondary mass spectrometry parameters are within the mass scanning range of m/z: 50 to 1250; Cluster removal voltage: 100 V; Collision energy: 40 eV; Collision energy swing: 20 eV	Fast and efficient, with high accuracy	[22]
	NIR-HSI		Camera exposure time: 3500 μ s<br>Height between lens and plate: 17.9 cm<br>Moving speed of conveyor belt: 13.8 mm/s	Simultaneously measure total phenols, total flavonoids, and total anthocyanins.	[23]
	CIRs and Si-ACO-PLS		Initial population: 95, maximum iteration count: 50, maximum decay count: 0.6, maximum cycle count: 10, variable selection threshold probability: 0.4	Simple, fast, and economical	[24]
	UPLC-MS	Chromatographic column: Agilent ZORBAX-SBC<br>Mobile phase: 0.1% formic acid, 0.1% formic acid acetonitrile<br>Positive and negative ion scanning	Flow rate: 0.8 mL/min, column temperature: 35C, detection wavelength: 254nm, injection volume: 5L.<br>M/z: 100-1500, atomization gas (GS1): 55 psi; Atomized gas (GS2): 55 psi; Curtain Air (CUR): 35 psi; Ion source temperature: 600 (positive)~550C (negative); Ion source	Simple operation	[25]

		mode	voltage: 5500 (positive)~4500 V (negative); Cluster removal voltage: 100 V; Focusing voltage: 10V		
Betaine	RP-HPLC	Chromatographic column: Hypersil NH2<br>Mobile phase: acetonitrile water (83:17, V/V);	Column temperature: 30℃, flow rate: 0.7 mL/min, detection wavelength: 195 nm	Good separation effect and strong reproducibility	[26]
	Ion-chromatography	Chromatographic column: Metrosep C cation analysis column<br>Rinsing solution: methanesulfonic acid solution<br>Detector: Conductivity detector	Column temperature: 38.5 ° C, flow rate: 0.8 mL/min;; Injection volume: 20 µL	Accurate and convenient	[27]
Tannin	UV	Ultraviolet spectrophotometer	670nm	Simple operation	[28]
Fatty acids	GC-MS	Dual flame ionization detector<br>Chromatographic column: HP-88 column		Convenient detection, good separation effect, high sensitivity, and low detection limit	[29]
Phenylpropanoid Derivatives	HPLC	Chromatographic column: open silica gel CC<br>Mobile phase: EtOH − H_2O, $CHCl_3$ − MeOH		High separation efficiency, fast speed, easy operation, and good detection sensitivity	[30]
Polyphenol glycosides	UPLC-QTOF-MS	Chromatographic column: COSMOSIL C18, reverse phase Kromasil 100-5C18, SH-Rxi-5Sil MS capillary<br>Mobile phase: 0.1% (v/v) acetic acid	Column temperature: 35 ℃, flow rate: 0.8 mL/min, 2.0 mL/min.	Accurate detection, fast and efficient	[31]

Table S3. Biological activity of LRM extract

Activity	Plant extract/compound	Dose/concentration	Experimental model/results	References
Anti-tumor	homogeneous polysaccharide LRP1-S2	7.45 µM, 14.9 µM	It has obvious inhibitory effect on the proliferation of three pancreatic cancer cells AsPC-1, BxPC-3 and PANC-1	[8]
	Anthocyanins	200 µg/m L	Increase the apoptosis rate of human liver cancer HepG2 cells, significantly inhibit the mRNA expression of JAK2 and STAT3 ($P<0.05$), and significantly inhibit the protein expression of p-	[32]

			JAK2 and p-STAT3 (P<0.05); Significantly increased mRNA expression of LC3 and protein expression of LC3- Ⅱ （P<0. 05)	
		12.5, 25, 50 , 100 μg/m L	The activity of human liver cancer HepG2 cells was significantly reduced (P<0.05)	[33]
		LRP (150 μg/ml), LRAC (20 μg/ml)	The mixture of LRP and LRAC can significantly inhibit the proliferation of tumor cells within 24 hours	[34]
		IC50 = 601.97 μg/mL<br>IC50 =445.79 μg/mL	LRM anthocyanin pt3g can inhibit the proliferation of prostate cancer LNCaP and PC-3 cells and promote cell apoptosis	[35]
		IC50 =361.58 μg/mL、IC50 =601.97 μg/mL 、 IC50 =445.79 μg/mL	LRM anthocyanin Pt3G can inhibit the proliferation of prostate cancer DU-145, LNCaP, and PC-3 cells by inducing cell apoptosis(p<0.05)	[36]
	Flavonoids	IC50 =110.49 μg/mL	Inhibition of Hep G-2 cell growth(P<0.05)	[37]
Prebiotics	LRP	100 mg/kg	The weight of mice was lower than that of the CT group (P<0.05),<br><br>The number of Lactobacillus and Bifidobacterium in the gut of HFA mice increased compared to the CT group, while the number of Enterobacterium and Enterococcus decreased (P<0.01)<br><br>The sIgA content in the intestinal mucosa of HFA mice was significantly higher than that of the CT group (P<0.01)	[40]
		6 mg/mL	The number of live bacteria in the experimental group was lower than that in the positive control group (P<0.01) and better than that in the blank group (P<0.05)	[38]
		0.5 g/L	Significantly promoted the growth of G2 (P<0.05)	[39]
		1.5 g/L	The free radical scavenging rates of G2 DPPH increased by 300.0% and 243.6%, respectively, and the reducing power increased by 6.7% and 4.7%; The free radical scavenging rates of L9 DPPH increased by 18.7% and 116.7%, respectively, and the reducing power increased by 4.1% and 4.1%	
	Anthocyanins	0.8% aqueous solution of anthocyanins	Increased diversity of gut microbiota in mice and altered the community structure of gut microbiota(P<0.05)	[41]
Anti-fatigue	LRP	0.5 g/mL	Can significantly increase the exhaustion time of swimming(p < 0.05)<br>LDH, IL-6, IL-1β, IL-2 were significantly decreased(p < 0.05)	[43]
		0.05 g/mL	Can significantly increase the exhaustion time of swimming(p < 0.01)<br>LDH(p < 0.05), TNF-α(p < 0.05), IL-6, IL-1β, IL-2 were significantly decreased, and SOD was significantly increased(p < 0.01)	
		0.10 g/mL	Can significantly increase the exhaustion time of swimming(p < 0.001)<br>LDH(p < 0.01), TNF-α(p < 0.05), IL-6, IL-1β, IL-2 were significantly decreased, and SOD was significantly increased(p < 0.001)	

		0.15 g/mL	Can significantly increase the exhaustion time of swimming(p < 0.001)<br>LDH(p < 0.01), TNF-α, IL-6, IL-1β, IL-2 were significantly decreased, and SOD was significantly increased(p < 0.001)	
Eye care	LRP	LMP: 500 μg/mL	Cell activity inhibited (P<0.05)	[44-45]
		LMP-1-1, LMP-3-1, LMP-4-1,the concentration of 25, 50, 100 μg/mL	Compared with the blank control group, the activities of SOD and GSH Px in ARPE-19 cells significantly decreased (P<0.01), while MDA significantly increased (P<0.01); Compared with the model control group, the activity of GSH Px and SOD significantly increased (P<0.01, P<0.05), while the content of MDA significantly decreased (P<0.05, P<0.01)	
		LMP-2-1: 25, 50, 100μg/mL	Compared with the blank control group, the activities of SOD and GSH Px in ARPE-19 cells significantly decreased (P<0.01), while MDA significantly increased (P<0.01); Compared with the model control group, the activity of GSH Px and SOD significantly increased (P<0.01, P<0.05), while the content of MDA significantly decreased (P<0.05, P<0.01)<br>The total apoptosis rate of ARPE-19 in the low and medium dose groups of LMP-2-1 was significantly reduced (P<0.05)<br>Compared with the blank control group, the model control group showed NLRP3, Caspase-1, IL-1 β Significant increase in expression (P<0.01, P<0.05); Compared with the model control group, the low and medium dose groups of LMP-2-1 showed NLRP3, Caspase-1, and IL-1 β Significant decrease in protein expression (P<0.01), NLRP3 and IL-1 in the high-dose LMP-2-1 group β The protein expression significantly decreased (P<0.01, P<0.05).	
Skincare effect	Anthocyanins	100ug/mL, 500ug/mL, 1000ug/mL	Compared with the blank group, the number of aging cells in HSFs significantly increased, with SA- β- The positive rate of Gal staining significantly increased (P<0.01). Compared with the UVB irradiation group, SA- β- The positive rate of Gal staining decreased (P<0.05)<br>The expression of aging genes p53 and p21 decreased with increasing drug concentration (P<0.05).	[47]
		0.1，0.5，1.0 mg/mL	Can significantly reduce HSFs and TNF- α The expression of (p<0.01).<br>TNF group treated with 1.0 mg/mL anthocyanins- α The most significant decrease in expression was observed (p<0.01).<br>Caspase-7 expression decreased while Survivin expression increased (p>0.05)	[48]
Nerve protection	LRP	5, 10, and 20 μM	Not causing significant cytotoxic effects	[50]
	Anthocyanins	50, 100, 200 μg/mL	Not causing significant cytotoxic effects<br>The significant decrease in ROS levels significantly increased the activity of CAT, SOD, and GPx (P<0.05)<br>Significantly reduced the activities of caspase-3 and bax, and significantly increased the activity of bcl-2 (P<0.05)	[51]
	Pn3G5G	100 mg/kg	Significant improvement effect on cognitive impairment in rats (P<0.01)<br>Significantly reduces neuronal apoptosis	[52]

			Significantly reduce AGEs, MDA, NF in D-galactose-treated rats- κ B. P65, TNF- α (P<0.05), COX-2 and IL-6 (P<0.01)	
Anti-inflammation	Anthocyanins	50mg/L	Compared with the normoxic group, the normoxic+AC group of rats had MDA and TNF in myocardial cells- α、 The content of IL-6 was significantly reduced (P<0.05), while the activities of CAT, HO-1, Na+- K+- ATPase, and Ca2+- ATPase were significantly increased (P<0.05). The activities of GSH Px, SOD, and IL-10 were all significantly increased (P<0.01);<br>Compared with the hypoxic group, the MDA and TNF in myocardial cells of rats in the hypoxic+AC group- α、 The content of IL-6 was significantly reduced (P<0.01), while the activities of CAT, GSH Px, SOD, HO-1, Na+- K+- ATPase, Ca2+- ATPase, and IL-10 content were significantly increased (P<0.01)	[53]
		238 mg / kg<br>59. 5 mg / kg	It can significantly reduce ankle joint swelling in rats at 6 and 24 hours after modeling (P<0.05) 01. P<0 05)<br>Significantly improved the limping gait of rats after 6 hours of modeling (P<0 05) High doses of AEL can continuously improve the limping gait of rats after 24 hours of modeling (P<0 05)	[54]
		100,, 200, 400 μg/ml	RASFS was significantly inhibited at 24 and 48 h (P<0.01).	[55]
Liver protection	LRP	30, 50, 150,200 mg/kg	The activities of SOD, CAT, and GSH Px were significantly increased, while the contents of MDA, AST, and ALT in liver tissue were significantly reduced (P<0.05)	[56]
	Proanthocyanins	140 mg/kg/d	Significantly reduced the accumulation of triglycerides in the liver, reduced inflammation, increased GSH Px activity, and decreased MDA levels.<br>The expression of the fatty acid oxidation related gene Scd 1 significantly increased, and the fatty acid synthesis related gene Ppar γ The expression of was significantly downregulated.	[57]
Bacteriostasis	Anthocyanins	MIC: 3.125 mg/mL	The best antibacterial effect on S.aureus	[58-59]
Protecting myocardial cells	Anthocyanins	50 μg/mL	LDH activity significantly decreased (P<0.05), cell density increased, cell boundaries were clear, and cell morphology was relatively normal<br>The fluorescence intensity of blue and red cells was significantly reduced, and the ROS content and apoptosis rate were significantly reduced (P<0.01). The mRNA and protein expression of Bax were significantly reduced (P<0.01), while the mRNA and protein expression of Bcl2 and Bcl2/Bax ratio were significantly increased (P<0.01)<br>The number of G0/G1 phase cells significantly decreased (P<0.05), while the number of S phase and G2/M phase cells significantly increased (P<0.05). The mRNA and protein expressions of CCNT2, CDK9, and FOXP1 were also significantly increased (P<0.01)	[62]
		50 μg/mL	The differentially expressed RNAs include 2980 mRNA (2365 upregulated, 615 downregulated), 101 miRNAs (62 upregulated, 39 downregulated), and 35 circRNAs (15 upregulated, 20 downregulated)	[63]
Hypoglycemic and hypolipemic	Anthocyanins	IC50=1.35 μg/mL	right α- Glucosidase activity has a significant inhibitory effect.	[65]

		IC50= 25.26 μg/mL	For Caco-2 cells α- Glucosidase activity also has a significant inhibitory effect	
		50 mg/kg<br>100 mg/kg	TG, TC, and LDL-C values significantly decreased, while HDL-C values increased (P<0.01).<br>The weight gain rate and liver index of SD rats were significantly reduced (P<0.05).<br>The concentrations of SOD and GSH Px both increased, while the concentrations of MDA decreased (P<0.01)	[66]
Anti-influenza	LRP	CC50=409.11 μg/mL<br>IC50=17.58 ± 0.38 μg/mL	It can inhibit the activity of A/H3 N2 virus	[67]
Anti-anxiety	Anthocyanins	3g/kg	The Conditioned place preference scores in the treatment group were significantly lower than those in the model group (P < 0.01).	[68]
Prolong life	LRM extracts	5、10 mg/mL	the middle (5 mg/mL)<br>and high doses (10 mg/mL) of LRM extracts significantly pro-<br>longed the average lifespan of C. elegans by 24.28% and25.19% (p< 0.05）	[69]
	LRP-S2	100, 500 mg/kg	LRP-S2 increased Alkaline phosphatase activity by 94.3% and showed the highest potential to stimulate osteoblast differentiation	[70]
	Anthocyanins	200, 400 mg/kg	Inhibition of ankle joint edema in mice began at 36 and 24 hours (both p<0.05), and showed the best inhibitory activity at 72 hours (all p<0.01).	[71]
Protecting mitochondria	Anthocyanins	50 , 100 μg/mL	Improved the vitality of DF-1 cells	[72]

Table S4. Product development of LRM extract

Product name	Sample	Process parameters	Advantage	References
Intelligent active packaging film	Anthocyanins, cassava starch, polyvinyl alcohol (PVA)	2% gelatinous starch solution, 2% PVA solution, 2% LRM anthocyaninss, 30% glycerol, temperature: 30 ℃, drying time: 30 minutes	Reduce the increasing environmental and health issues caused by traditional plastic packaging, and monitor food quality in real-time	[73]
Fibrous Membrane	Procyanidin	10% (W/V) gelatin acetic acid solution, 10% (W/V) polyethylene oxide acetic acid solution, 3% LRM procyanidin, spinning voltage: 40 kV, spinning rate: 100 mm/s; Spinning distance: 15cm; Environmental temperature: 20-30 ℃, relative humidity: 40%~60%	There is no heat involved in the spinning process, and the impact on bioactive substances is relatively small	[74]
Food packaging film	Cassava starch, Polyvinyl alcohol (PVA), LRM polyphenols	Cassava starch (20 g/L), PVA(20 g/L), starch and PVA solution: 1:1, 2% LRM polyphenols, 25% glycerol, temperature: 30 ℃, drying time: 48 hours.	It has antioxidant and antibacterial properties, as well as strong mechanical and barrier properties of the film.	[75]

Smart tags	Anthocyanins	LRM anthocyanins: 10mg/mL, soaking time: 2 minutes	Can provide real-time and accurate feedback on freshness information	[77]
PH sensitive membrane		ASKG solution and SPI solution: 1:1, 40% glycerol, room temperature drying time: 12 hours, hot air drying temperature: 50 ℃, drying time: 12 hours. 1%, 2%, 3%, and 4% (g/mL) acidified ethanol solution of LRM anthocyanins.	The pH sensitive membrane prepared has the advantages of high sensitivity, non toxicity, low cost, and biodegradability, and can be used for real-time monitoring of meat freshness.	[78]
Colorant		LRM anthocyanins: 25 μ Mol/L<br><br>0.06 M metal ion solution (Al 3+or Fe 3+), then in a pH 3-9 buffer (i.e., metal ion concentrations of 2.5, 12.5, 25, 50, 125, 250, 750, and 1500) μ Mix specific target anthocyanins and metal ion molar ratios (1:0.1, 1:0.5, 1:1, 1:2, 1:5, 1:10, 1:30, 1:60) with LRM anthocyanin extract in mol/L.	Has strong stability.	[79]
Accelerator		LRM anthocyanin addition amount: 0.06 mg/mL	Significantly enhance the antioxidant capacity of mycelium cells, increase biomass and polysaccharide production.	[80-81]
Chewable	Procyanidin	LRM procyanidin 40%, microcrystalline cellulose 15%, dextrin 20%, xylitol 10%, mannitol 10%, malic acid 2.5%	Smooth surface, uniform and consistent color, sweet and sour taste, smooth entrance, moderate hardness and brittle-ness.	[82]
	LRM	Crystal cellulose: 15 g, xylitol: 12.5 g, carboxymethyl cellulose sodium: 15 g, LRM: 41 g, water: 9 g, magnesium stearate: 13 g	Smooth surface, delicate and compact cross-sectional organi-zation, refreshing and delicious, with a harmonious sour and sweet taste, and a smooth entrance	[83]
		Material water ratio: 1:30 (g: mL), inoculation amount of 3% (volume fraction), fermentation temperature: 37 ℃, fermentation time: 15 hours	It has the unique flavor of LRM and the unique aroma of lactic acid fermentation, with a sweet and sour taste and a delicate taste; Organizational form: The product is clear and trans-parent, with good fluidity and no visible impurities or precipi-tates	[84]
Beverage	LRM	Pure milk: 77%, passion fruit juice: 5%, LRM juice: 11%, sucrose: 7%, thickener: 0.06%, bacterial inoculation amount: 0.003%, fermenta-tion time: 6.5 hours	It has certain antioxidant properties, good organizational state, no whey precipitation, no layering, and has the fra-grance of passion fruit and LRM, as well as the unique flavor of dairy beverages. The sour sweet ratio is appropriate	[85]
		Aronia melanocarpa (Michx.) Elliott: 20%, LRM40%, white sugar: 10%, citric acid: 0.10%, xanthan gum: 0.08%, carboxymethyl cellulose sodi-um: 0.06%, pectin: 0.04%	Rich in nutrients, unique in flavor, with antioxidant and other functions	[86]
		LRM: 1%, salt: 0.2%, citric acid: 0.1%, xylitol: 6%	The beverage is purple in color, clear liquid, sweet and sour, and has the aroma that LRM should have	[87]
		LRM: 8.20%, white sugar: 9%, bacterial powder: 0.91%, temperature: 42 ℃, fermentation time: 4.94 hours	Suitable color, well organized, uniform texture, sour and sweet taste, with a unique taste and aroma of LRM	[88]
Fruit vinegar	LRM	Strain: Winecoideryeast, LRM: 15%, initial sugar content: 17%, fer-	Maximizing the preservation of LRM's original flavor, nutrition,	[89]

		mentation temperature: 32 ℃, inoculation amount: 4%; Strain: Shanghai Brewing 1.01 Acetobacter, fermentation temperature: 30 ℃, inoculation amount: 7%, liquid content: 42mL/250mL.	and health benefits, adding the unique mellow flavor to fruit vinegar	
Microcapsule	Anthocyanins	Embedding time: 66 min, CS: 0.2 mg/mL, HP- β- CD: 1.93 mg/mL.	Improving the pH and thermal stability of LRM anthocyanins	[90]
Dyeing agents	LRM pigment	80% ethanol/water mixed solution, solid-liquid ratio: 1:10, staining temperature: 80 ° C, pH=2	Dyed wool fabrics can achieve higher surface depth, and their antioxidant and antibacterial properties are stronger.	[91]
